# Engineering Aging: Approaches to Model and Deconstruct Biological Complexity

**DOI:** 10.1002/adma.202512523

**Published:** 2025-09-09

**Authors:** Habib Joukhdar, Sunny Shinchen Lee, Thomas R. Cox, Yu Suk Choi, Steven G. Wise, Jennifer L. Young, Giselle C. Yeo, Khoon S. Lim

**Affiliations:** ^1^ School of Medical Sciences University of Sydney Sydney NSW 2006 Australia; ^2^ Sydney Biomanufacturing Incubator University of Sydney Sydney NSW 2006 Australia; ^3^ School of Life Sciences University of Sydney Sydney NSW 2006 Australia; ^4^ Charles Perkins Centre University of Sydney Sydney NSW 2006 Australia; ^5^ Garvan Institute of Medical Research Sydney NSW 2010 Australia; ^6^ School of Clinical Medicine St Vincent's Clinical Campus UNSW Sydney Sydney NSW 2052 Australia; ^7^ School of Human Sciences University of Western Australia Perth WA 6009 Australia; ^8^ Mechanobiology Institute National University of Singapore Singapore 117411 Singapore; ^9^ Department of Biomedical Engineering College of Design and Engineering National University of Singapore Singapore 117583 Singapore

**Keywords:** aging, biomaterials, biofabrication, tissue models

## Abstract

The disparity between the global increase in life expectancy and the steady decline in health outcomes with age has been a major driver for developing new ways to research aging. Although this current tools for studying aging outside of the human body—such as animal models and cells in a dish—have improved this fundamental understanding of the markers and key mechanisms underlying this process, several limitations remain. Animal models are poor biological representations of humans and have a weak track record of translating pre‐clinical results into successful clinical applications. Similarly, current 2D cellular models do not recapitulate the dynamic 3D environment of human tissue. This gap between the need for accurate biological mimicry and the limitations of current aging models presents an exciting opportunity for the field of biofabrication. Over the past decade, the combination of biofabrication and advanced biomaterials has shown potential to engineer high‐resolution features that change over time or respond to specific stimuli. In this perspective, the current state of in vitro aging models is reflected, identify the key features that new models must emulate, discuss the technologies available to meet these complex specifications, and consider some of the potential challenges facing the field.

## Graphical abstract

The macro‐experiences of aging are the result of an accumulation of micro‐changes that occur over time.

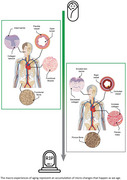

## 1 Introduction

According to World Health Organization data aggregates, the global average life expectancy has increased by ≈5 years over the last two decades.^[^ ^1^ ^]^ However, while the average life expectancy for individuals over 60 is ≈20 years, they can expect to be in full health for only ≈15 years.^[^ ^2^ ^]^ This disparity in lifespan and healthspan poses a continuing challenge, as longer life expectancies exert increasing strain on healthcare systems. In response to these challenges, the fundamental science of biological aging has become the focus of a large body of literature and organizations like the International Cell Senescence Association,^[^ ^3^ ^]^ the SenNet Biomarkers Working Group^[^ ^4^ ^]^ and an international coalition of academic institutions. These efforts have led to key breakthroughs in understanding the cellular mechanisms of aging, made possible by in vivo and in vitro models that enable exploration of aging‐related cellular mechanisms.

Aging models are designed to probe the fundamental biology of aging and to explore potential treatments for combating aging and age‐related diseases. These in vitro and in vivo models have significantly enhanced our understanding of aging as a whole and cellular senescence. Cellular senescence, or cellular growth arrest, may imply aging, but it can also be a response to stressful stimuli, such as injury.^[^ ^5^ ^]^ Key findings of in vitro and in vivo studies include identifying hallmarks of aging, such as molecular markers of cellular senescence (e.g., p53/p21^CIP1/WAF1^ and p16^Ink4a^/Rb)^[^ ^6^ ^]^ and in vitro biochemical tests for senescence (e.g., staining for senescence‐associated beta‐galactosidase at pH 6).^[^ ^6^, ^7^ ^]^ Furthermore, technologies such as next‐generation sequencing and liquid chromatography‐mass spectrometry (LC‐MS) have unlocked transcriptomic and proteomic understanding of cellular aging, by providing in‐depth libraries of aging‐related markers at the gene and protein levels.^[^ ^6^ ^]^ Online databases curated through international efforts, such as SENCAN (a cancer senescence classifier/database),^[^ ^8^ ^]^ SenMayo (organ and tissue‐specific senescence gene sets),^[^ ^9^ ^]^ and SenePy (transcriptomic signatures of in vivo tissue‐specific senescence),^[^ ^10^ ^]^ have unveiled the landscape of cellular senescence in healthy aging and disease across multiple tissues and species.

In vivo models are the current gold standard for aging studies. In vivo models include invertebrate (e.g., worms, Drosophila) and vertebrate systems (e.g., mice, rats, and primates). These non‐human models comprise fully functional organ systems, allowing researchers to ask questions holistically and execute experiments within a complex biological environment. However, these models vary substantially from human physiology, leading to poor prediction of clinical outcomes. This dissonance between animal models and human systems is demonstrated by the low drug success rate, whereby only ≈5% of drug candidates tested on animal models successfully pass clinical trials.^[^ ^11^ ^]^ On the other hand, in vitro models to study cellular aging mainly consist of cells grown on 2D tissue culture plastic (TCP),^[^ ^6^, ^12^ ^]^ which trades off biological complexity for scalability and tunability. Such systems provide researchers with control over critical variables, including the cell type (or types) and the use of extracellular biochemical components as a surface coating (e.g., gelatin and Matrigel®). Furthermore, TCP is backed by a suite of equipment that has been used to streamline analysis and automate execution. However, like animal models, TCP significantly differs from native human tissue, in which cells are physically organized within an extracellular matrix (ECM) that varies in form and function depending on the tissue origin. For example, healthy myocardium has a stiffness of ≈10 kPa and composed of cardiomyocytes arranged in aligned fibers.^[^ ^13^ ^]^ In contrast, TCP has local stiffness properties within the gigapascal range and is a uniform 2D environment, significantly different from how cells occupy space within 3D tissue.

**Figure 1:**
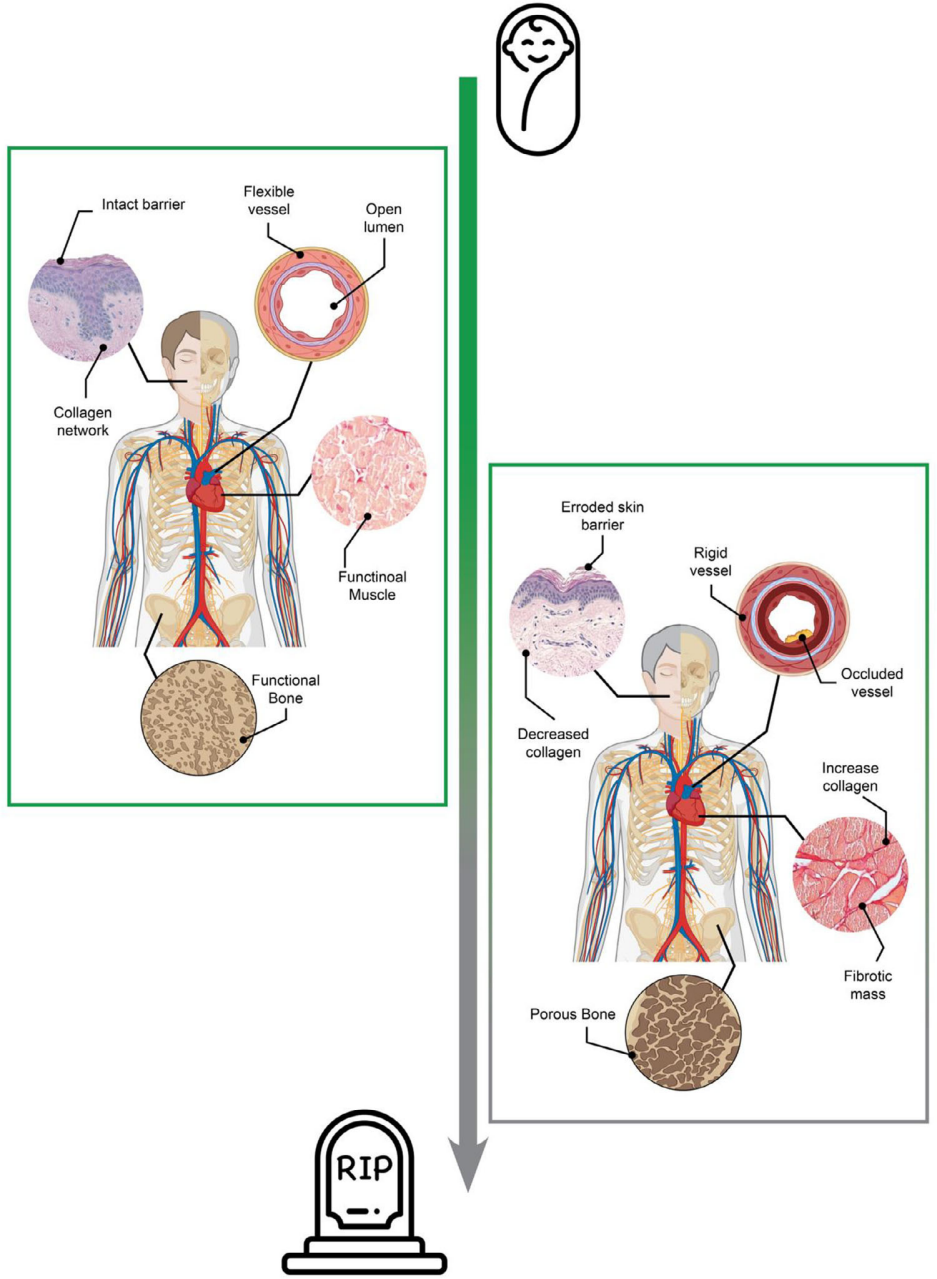
Illustrating the microphysiological changes of aging that occur over time. The macro‐experiences of aging represent an accumulation of micro‐changes that happen as we age. Skin loses collagen, becoming less elastic and more wrinkled. Arteries stiffen and narrow, accumulating plaque and debris over time. Cells in cardiac and skeletal muscle transition into a fibrotic state, losing functional cell mass and becoming stiffer over time. Bone becomes more brittle due to conditions like osteoporosis. Reproduced under the terms of the Creative Commons CC‐BY License.^[^ ^15^ ^]^ Copyright 2011, Aging and Disease. Reproduced under the terms of the Creative Commons CC‐BY License.^[^ ^16^ ^]^ Copyright 2022, John Wiley & Sons.

Aging is, at both the organismal and cellular scales, a dynamic physical process combined with functional deterioration and loss of regenerative capacity over time (**Figure** **1**). The macroscale manifestations of age (e.g., stiffer joints, occluded vessels) are an accumulation of microscale events occurring on the cellular and tissue level in response to the extracellular environment, and these phenomena need to be replicated in a new generation of in vitro aging models.^[^ ^12^ ^]^ This gap can be addressed by applying a new focus on biomimicry. Biomimicry, as the foundation of regenerative medicine, is also core to the modelling of disease states and natural biological processes such as aging. In a structural sense, physically relevant biomimicry is built on recapitulating the properties of the ECM. The ECM provides cells with a physical and chemical environment that regulates cellular mechanisms. Engineering biomimetic physical environments from readily available, off‐the‐shelf materials (e.g., silk fibroin, alginate, agar, and gelatin) through controllable fabrication techniques (e.g., 3D printing) is a critical objective in regenerative medicine.^[^ ^14^ ^]^ Biomaterial development is based on the understanding that a cell's physical environment is essential for maintaining cell health, facilitating cellular movement, directing cellular differentiation, and even dictating the host immune response to an implanted material. Combining the extensive characterization tools developed for in vitro and in vivo models with advances in the fabrication of biomimetic physical environments is the logical next step in executing a new generation of physiologically relevant in vitro aging models.

Recent breakthroughs in off‐the‐shelf biofabrication tools have enabled the engineering of high‐resolution features (e.g., down to a micron) or dynamic physical cues that change over time, or remodeled due to extrinsic researcher‐activated stimuli (e.g., different light wavelengths).^[^ ^17^ ^]^ These microfabrication techniques can be used to design complex physiologically relevant models with biomimetic features (e.g., aligned fibers in cardiac tissue)^[^ ^18^ ^]^ that change over time or in response to different user‐triggered stimuli (e.g., light‐activated stiffening or softening).^[^ ^19^ ^]^ The demand for more biomimetic aging models is further strengthened by the global movement toward phasing out animal testing, making the need for progress in this field particularly timely. As capabilities within regenerative medicine continue to evolve, the focus will likely shift from process development to the execution and validation of refined models that closely mimic the dynamic nature of human aging. This perspective highlights some of the most recent developments regarding aging models, the tissue‐specific aspects they must emulate to achieve higher accuracy and relevance, and their current limitations. These findings are also contextualized within the current state of market‐ready technologies that can provide pathways to add new levels of functional biomimicry to these models. We also discuss potential opportunities and challenges related to advanced characterization systems and novel personalized medicine platforms.

## 2 The Dynamic Environment of Aging – What Properties Need to be Emulated?

Biomimetic in vitro models aim to accurately replicate both healthy and diseased tissues in a 3D microenvironment. Accordingly, the ideal in vitro aging model would transition from mimicking healthy, young tissue to aged tissue over time.^[^ ^20^ ^]^ Capturing this progressive shift is crucial, as one of the primary goals of aging studies is to investigate how cell health evolves as the host environment changes with age. In addition to engineering this temporal shift, a significant challenge lies in aligning model specifications with the unique aging physiology of the target tissue. Different parts of the body age in various ways in relation to the physical properties; some become stiffer,^[^ ^21^ ^]^ while others become softer,^[^ ^22^ ^]^ more brittle or weaker.^[^ ^23^ ^]^ It is important to highlight that engineering a new class of aging model is also a conversation in engineering complexity at the physiological and temporal scale. From a temporal perspective, the most accurate template to emulate will be that of an aging human being, an experimental timeline that is challenging and impractical. Therefore, the next generation of dynamic aging models should embody a form of simplified complexity, whereby key aging events are physiologically captured in the model at an accelerated timeline. Biomaterial‐based in vitro aging models have demonstrated this record of accelerated timeline, whereby researchers identified cellular senescence within 3,^[^ ^24^ ^]^ 9,^[^ ^13^ ^]^ and 120^[^ ^25^ ^]^ days of culture on biomaterials that do not change with time. Targeting these events starts with understanding the tissue‐specific physiology of the aging ECM, thus setting a specification sheet for model design and engineering.

### 2.1 The impact of Age on the Physical Properties of Aligned Soft Tissues

Cellular alignment is crucial for activating unique functions across various tissues.^[^ ^26^ ^]^ In load‐bearing structures, such as bone, and physically demanding tissues like cardiac and skeletal muscle, the aligned distribution of cells enhances durability and strength. Additionally, interlinked aligned cells support the rapid transmission of electrical signals, as seen in neurons and cardiomyocytes, facilitating quick reflex responses and synchronized actions. With age, the loss of alignment becomes evident in these tissues.^[^ ^27^ ^]^ The progressive loss of aligned regions at the microscale, driven by age‐related ECM functional deterioration, leads to macroscopic consequences, affecting biological processes like tissue durability, range of motion, and cognitive abilities.

**Figure 2:**
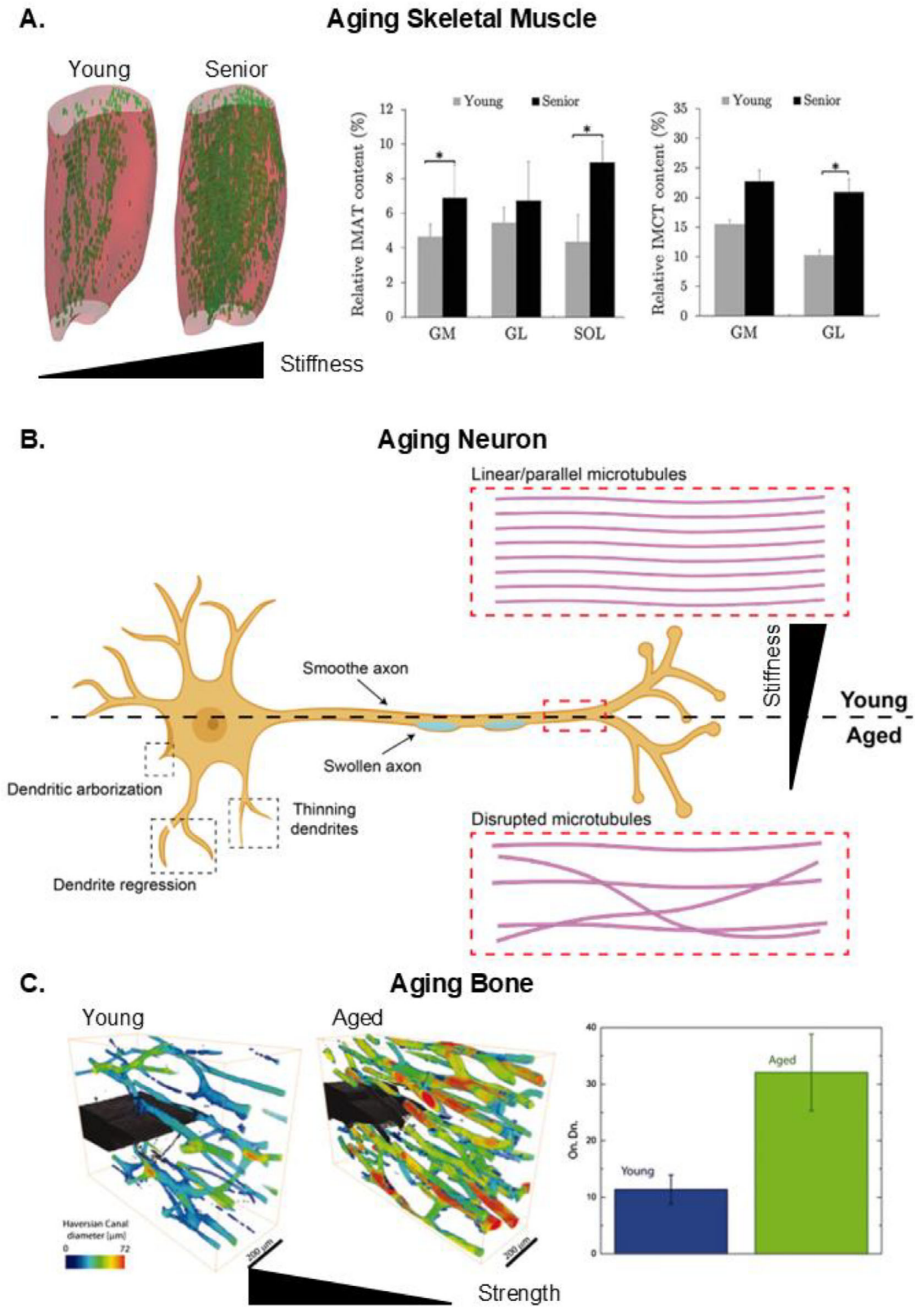
Microscopic physical changes of aging tissue. A) Functional muscle mass decreases with age and is replaced by a growing percentage of intramuscular adipose tissue (IMAT) and intramuscular collagen tissue (IMCT). The degree of physical changes depends on the muscular region. Abbreviations are gastrocnemius medial (GM), gastrocnemius lateral (GL), and soleus (SOL). B) Aging neurons lose dendritic anchor points over time (i.e., arborization), dendritic thinning, and swelling. As people age, microtubules within the axon begin to degrade and misalign, resulting in swelling and softening of the axon. C) The harversian canal (a functional unit of bone) increases in density and size over time. This increase is a significant contributor to the fragility of bone with age. Reproduced under the terms of the Creative Commons CC‐BY License.^[^ ^28b^ ^]^ Copyright 2020, The Authors, published by Frontiers. Reproduced under the terms of the Creative Commons CC‐BY License.^[^ ^29^ ^]^ Copyright 2023, Wolters Kluwer Medknow Publications. Reproduced (Adapted) with permission.^[^ ^30^ ^]^ Copyright 2011, National Academy of Sciences.

As the body ages, cells in skeletal muscle transition into a fibrotic state (**Figure** **2**A).^[^ ^28^ ^]^ The increased deposition of disoriented collagen raises overall tissue stiffness, negatively impacting the differentiation potential of satellite cells to form myotubes. Consequently, the continuous rise in ECM stiffness inhibits the formation of functional muscle units, leading to a decrease in muscle mass and eventual loss of muscle function or performance. MRI scans that quantify muscle density show that in senior citizens, regardless of muscle region, the mean intramuscular adipose and collagen content increases by ≈4%–8% and 8%, respectively.^[^ ^28b^ ^]^ A separate study quantified ECM content in muscle at different ages and showed that ECM content increased from 3.3% in young patients to 8.2% in older patients.^[^ ^27^, ^28^ ^]^ Similar to skeletal muscle, the functional loss of aligned neural cells is a major cause of neurodegenerative disease. However, the primary trigger for physical changes in neural tissue is cellular degradation, rather than a change in ECM physical components (Figure 2B).^[^ ^29^ ^]^ Dendritic length shortens with age by 13% to 76%, depending on the location of the dendrite.^[^ ^29^ ^]^ Shortening of dendrites and loss of actin tubules cause neural tissue to soften with age,^[^ ^22^ ^]^ in contrast to the stiffening observed in other aligned tissues such as cardiac and skeletal muscle.

These age‐related changes in tissue architecture have profound implications beyond functional decline. The loss of organized matrix structure generates microenvironments that are increasingly permissive to cancer initiation and progression. Disrupted collagen alignment and increased matrix stiffness can promote cancer cell migration, while senescent cell‐derived inflammatory factors drive malignant transformation in neighboring epithelial cells. Understanding these aging‐cancer interactions through engineered models could reveal new therapeutic approaches for the predominantly elderly cancer patient population.

### 2.2 The Impact of Age on the Physical Strength of Bone

The strength and durability of tissues, such as bone, change with age. ≈30% of falls among people over 60 in the US result in moderate or serious injuries, making the elderly the highest risk group, as quantified by the WHO.^[^ ^31^ ^]^ Interestingly, while the stiffness of bone does not drastically change with age,^[^ ^31^, ^32^ ^]^ its overall strength significantly decreases with each passing decade. This increase in fragility is due to decreased bone density^[^ ^23^, ^30^ ^]^ and reduced bone healing capacity over time.^[^ ^30^, ^33^ ^]^ Healthy bone comprises mineralized collagen molecules and hydroxyapatite nanocrystals,^[^ ^30^ ^]^ and its hierarchical organization and densification define its physical properties. Although loss of bone density and increased porosity negatively impact bone durability and strength, evidence shows that bone resilience deteriorates in aging patients even if bone density is maintained. This decline is likely due to a progressive increase in non‐enzymatic collagen crosslinking and an overall increase in osteon density, which negatively impacts bone plasticity (i.e., toughness) (Figure 2C).^[^ ^30^ ^]^ Over time, the loss of flexibility in the bone ECM increases the cumulative impact of microcracks from repetitive strain or daily wear and tear, making older individuals more susceptible to bone fractures.

## 3 Current State of the Art of Aging Models

### 3.1 In vivo Models for Aging Fall Short of the Mark

Current in vivo models have been essential for enhancing our understanding of aging and senescence, yet they have three significant shortcomings: ethics, translatability, and sustainability. Notably, animal experimentation is often criticized for yielding poor results that are not translatable. The transition from therapeutics tested on animals to those successfully deployed in the market has a success rate ranging from 5%^[^ ^11a^ ^]^ to 8%.^[^ ^11b^ ^]^ This low success rate intensifies ethical concerns and the practicality of in vivo animal models. Furthermore, animal models have a large carbon footprint and place significant resource, space, and financial demands on research infrastructure. The disposal of animal carcasses and associated consumables (e.g., syringes, bedding, cages) is a major pollutant.^[^ ^34^ ^]^ Animals require housing in specialized facilities and maintenance by staff over timespans that can range from months to years, depending on the animal's lifespan and the nature of the experiment.^[^ ^34b^ ^]^ Although standard in vitro lab practices rely on animal‐sourced components (e.g., Matrigel, cell culture serum, collagen), efforts are underway to develop and deploy animal‐component‐free versions. Animal product alternatives such as Econik's recombinant collagen (VECOLLAN®)^[^ ^35^ ^]^ or Media City Scientific's^[^ ^36^ ^]^ synthetic alternative to fetal bovine serum (a popular ingredient in cell culture) are either on the market or on the verge of hitting consumer shelves. Furthermore, leading lab supply brands such as Corning^[^ ^37^ ^]^ and Thermo Fisher^[^ ^38^ ^]^ are developing sustainable lines of TCP products. Some private industries are even collaborating with international nonprofits such as the My Green Lab initiative to inform on eco‐friendly lab practices and sustainable supply chains.^[^ ^39^ ^]^

### 3.2 Tapping into the Potential of in Vitro Aging Models

The ECM is one of the most complex and nuanced features of our body. It has two main components: A biochemical component and a biophysiological component.^[^ ^40^ ^]^ The biochemical component consists of a mix of cell‐adhesive ligands, matrix‐bound growth factors, cytokines, and other signalling molecules produced by resident cells, as well as ECM degradation products.^[^ ^40a^ ^]^ These biochemical components regulate biological processes that impact phenomena such as cell differentiation,^[^ ^41^ ^]^ mechanisms surrounding cell maintenance (e.g., autophagy),^[^ ^42^ ^]^ and other biological functions crucial for cellular function. The biophysiological component serves as the biological equivalent of a living scaffold.^[^ ^41^ ^]^ As a scaffold, the ECM provides cells with structural support and arranges them in a physical environment that is complementary to the biological function of the host tissue.^[^ ^26b^ ^]^ Furthermore, the biophysical interaction between ECM and cells has also been identified as a key player in controlling cell fate via mechanotransduction‐driven pathways.^[^ ^43^ ^]^ An example of this is aligned tissue, such as skeletal muscle and certain features of neuronal cells (e.g., axons).^[^ ^29^ ^]^ The physical alignment of these tissues complements the need for load‐bearing functions,^[^ ^26b^ ^]^ rapid electrochemical signal transduction throughout the body,^[^ ^26a^ ^]^ and biomechanical (i.e., mechanotransduction) signalling.^[^ ^43a^ ^]^ Like any other component of our body, the ECM is dynamic; it develops as we mature into adulthood and eventually functionally deteriorates with age (e.g., stiffer muscles, more fragile bones), leading to a loss of function and the features of aging that we have come to know.^[^ ^22^, ^27^, ^28^, ^40^ ^]^

ECM has also been a focus in several in vitro aging studies because of its critical role in cell biology,^[^ ^40a^ ^]^ its relatively accessible nature for analysis,^[^ ^26^, ^44^ ^]^ and the fact that it changes with age due to time and the overall longevity and stability of many secreted ECM molecules.^[^ ^27^, ^28^, ^40^ ^]^ The scope of in vitro ECM studies ranges from examining the effects of individual ECM components on aging^[^ ^45^ ^]^ to assessing the overall impact of aged ECM on cellular health and aging.^[^ ^40b^ ^]^ For example, Lee et al. recently reported the potential protective properties that tropoelastin, a precursor to elastin (an ECM component), has on the senescence progression of mesenchymal stem cells (MSCs).^[^ ^45^ ^]^ To investigate the effects of the whole ECM on aging, researchers harvest decellularized ECM (dECM) from tissues donated by patients or explanted from animals of various ages, to engineer representative environments that capture patient‐specific ECM at specific life stages.^[^ ^46^ ^]^ This approach allows researchers to assess questions related to whether young dECM protects cells from aging, such as if exposing aged cells to young dECM will promote the rejuvenation of aged cells, or if exposing young cells to aged dECM will prematurely “age” them.

In the space of non‐TCP or ECM in vitro aging models, stiffness is the most dominantly studied feature of aging. These models have been made out of silicone,^[^ ^24^ ^]^ modified natural polymers such as gelatin methacryloyl (GelMA),^[^ ^14^, ^25^, ^47^ ^]^ and synthetic polymers such as polyacrylamide,^[^ ^13^ ^]^ engineered to match the physical properties of a specific tissue at different ages. For example, models targeting skeletal or cardiac muscle considered stiffnesses of 5–10 kPa (young), 10–30 kPa (middle‐aged), and >100 kPa (old),^[^ ^13^, ^25^, ^47^ ^]^ while cartilage models would target stiffnesses ranging from 180 kPa (healthy) to 750 kPa (aged‐unhealthy) and 1500 kPa (pathological).^[^ ^24^ ^]^ These studies show that cells grown in stiffer environments are likely to express senescence phenotypes or markers earlier in their cellular lifespan. However, consolidating physical parameters against clinical data will be an essential consideration. For example, a cross‐sectional study led by Villemain et al^[^ ^48^ ^]^ used non‐invasive shear wave imaging to estimate the myocardial stiffness of a total of 60 healthy patients aged between 20 and 79 years. Villemain et al.*’*s^[^ ^48^ ^]^ study clinically showed that myocardial stiffness increases with age from 2.59 ± 0.58 kPa for young (20–39 years), to 4.70 ± 0.88 kPa for middle‐aged (40–59 years) and to 6.08 ± 1.06 kPa for old (60–79 years). Although this study demonstrated the progressive stiffening of aging myocardial tissue, the values presented are in contrast with some values highlighted in this discussion. In contrast to an “ideal” stiffness value, some studies have demonstrated that cardiomyocytes grown in vitro function best on surfaces ∼10kPa,^[^ ^13^, ^49^ ^]^ ≈1.7 times the value of data from live patients. The discrepancies in values are likely due to differences in tissue sources, sample preparation, and measurement protocols. Therefore, demonstrating the need to be mindful of design parameters for tissue‐specific in vitro models.

Moreover, these studies overlook the spatiotemporal dynamic nature of ECM. Aging is an irreversible process that progresses from maturation in youth to regression till eventual death. While these studies address age‐associated changes by growing cells on materials with varying stiffnesses to represent different ages, they do not model transitional effects – what occurs when healthy cells are grown in an environment that gradually stiffens or softens over time? Additionally, living organisms occupy a 3D space, yet most aging studies frame their inquiries based on models in which cells are grown on a 2D surface. This point is critical, as cells behave differently depending on which environment they're in. Cells might spread when seeded on stiff (20‐30 kPa) surfaces, but shrink when encapsulated in materials of the same stiffness.^[^ ^50^ ^]^ Cells grown on homogenous 2D surfaces will be uniformly distributed, but cells grown on^[^ ^26^ ^]^ or encapsulated within^[^ ^18^, ^51^ ^]^ aligned morphology will grow in the direction of the engineered micro‐topological features. While some 3D approaches have encapsulated cells in hydrogels, the uniform porosity of such systems limits their biomimicry to tissues with a similar structure (e.g., cartilage).^[^ ^51^, ^52^ ^]^ Tissues with more complex geometries and/or hierarchical distribution of cells (e.g., bone, skin, cardiac, or skeletal muscle) require architectural parameters to be engineered with spatial precision.^[^ ^26a^ ^]^ Regardless of tissue class, cellular architecture is important for function, complementing the unique physical functions (e.g., aligned architecture to support load‐bearing tasks or pump blood throughout the body) of the tissue under study.

### 3.3 Organ‐on‐a‐chip as an Aging Model

Organ‐on‐chip (OoC) is an advanced form of in vitro modeling that combines the engineering‐based tunability of TCP with the organ systems advantage of in vivo models.^[^ ^53^ ^]^ OoC emulates human physiology by culturing cells in or near microchannels.^[^ ^53^ ^]^ OoC models can be engineered to physiologically represent a specific organ or tissue, act as a single independent unit, or assembled into a series of OoCs to represent an entire organ system on a chip.^[^ ^53^ ^]^ OoC are also compatible with non‐invasive screening technology and continuous culture conditions, making it a potentially high‐impact model for long‐form culture in vitro therapeutic screening.^[^ ^54^ ^]^ OoC also allows remarkable flexibility in the choice of materials for channels, flow rates, cell types, and even the complexity of the channels themselves.^[^ ^53^, ^55^ ^]^ In the context of aging, OoCs have primarily been used to model aging‐related diseases.^[^ ^56^ ^]^ Flow‐related phenomena, such as blood vessel stenosis or stiffening, serve as excellent case studies for OoC. Channels can be custom‐engineered to match the physical dimensions and stiffness of various microchannels (e.g., arterioles and capillaries), flow can be optimized to replicate natural rates, and the cells in these channels can be hand‐selected to model diseased or healthy vessels.^[^ ^57^ ^]^ Additionally, artificial obstructions or pathways can be engineered into these channels to induce occlusion or stenosis.

The tuneability and adaptability of organ‐on‐chip (OoC) systems allow them to be mostly compatible with standard laboratory instrumentation (e.g., confocal microscopy)^[^ ^58^ ^]^ and custom measurement equipment (e.g., oxygen sensor and regulator).^[^ ^59^ ^]^ These devices can be designed to conform to the planar dimensions of glass slides or specific wells of standard TCP, enabling seamless integration with existing analytical tools. Although OoCs can be designed to match the dimensions of standard microscope mounts, the OoC might be contained within several mm‐thick plastic. The casing dimensions may place the channel outside the range of working distance of high magnification of objectives, and thus adding another workflow consideration when engineering OoC models.^[^ ^58^ ^]^ Furthermore, most initial OoC platforms were fabricated using silicone‐based materials such as PDMS, which posed specific limitations such as unintended absorption of proteins and small molecules, and difficulty in cleaning narrow microchannels, making these systems generally limited to single‐use applications.^[^ ^53^ ^]^ PDMS can even absorb fluorescent stains used to tag specific cellular features, potentially impairing the analytical workflow.^[^ ^60^ ^]^ However, recent advancements in OoC technologies have incorporated cutting‐edge biofabrication techniques. Modern 3D bioprinters, capable of achieving micron‐scale resolution, now allow for the fabrication of cell‐laden hydrogel‐based OoC with enhanced biomimicry, capable of capturing certain aspects of physiological relevance (e.g., integration of growth factors and other chemokines into the hydrogel matrix) of native tissues.^[^ ^55^, ^61^ ^]^

## 4 Future Directions in Engineering Novel in Vitro Aging Models

### 4.1 Harnessing Advanced Manufacturing to Engineer a New Class of Physical Aging Models

Engineering models of aging involve recreating the physical features of tissue‐specific ECM.^[^ ^22^, ^26^, ^28^, ^44^ ^]^ For instance, the ECM of heart valves consists of dense fibrous networks with aligned microstructures and fibers several microns in diameter.^[^ ^62^ ^]^ In contrast, skeletal muscle and nervous tissue feature aligned tubules with dimensions ranging from tens to a hundred microns, while bone comprises a dense mineralized framework with pore features in the millimeter range.^[^ ^62^ ^]^ Crafting these structures requires a synergistic approach combining material design and a fabrication workflow that exceeds the sum of its parts. Decades of effort in merging advanced biofabrication (e.g., 3D bioprinters) equipment with material design have transitioned from custom in‐house solutions to versatile, off‐the‐shelf, market‐ready fabrication machines (**Table** **1**) capable of engineering features down to the submicron level. The products and technologies listed in Table 1 are supported by an extensive library of biomaterials from companies like BioINX and CELLINK, optimized for these and similar fabrication platforms.

**Table 1:** Commercially available 3D bioprinters.

Technology	Description	Key Innovation	Manufacturer
Volumetric 3D printing	Printing by projecting an entire model into a photocurable ink, rather than layer‐by‐layer like traditional printers.	Rapid (<1 min) high‐resolution (≈25 µm) printing of whole structures.^[^ ^63^ ^]^	Readily 3D, Xolo3D
Two‐photon polymerization	Curing a photocurable resin by applying two focused beams of light at a single point.	Can achieve submicron resolution in soft‐biomaterials (e.g., GelMA^[^ ^64^ ^]^)	Nanoscribe, Upnano
Filamented light (FLight) 3D printing	Projecting a beam of light with varied light intensity into a photocurable resin to extrude aligned fibers.	A non‐cytotoxic approach to engineer aligned fibrosity with pore sizes ranging from 2‐30 µm into fully hydrated hydrogels^[^ ^18^ ^]^	Readily 3D
Sound‐induced morphogenesis	Patterning single cells, spheroids, or particles using acoustic waves.	Rapid low‐shear patterning of living cells or tissue in biomaterials.^[^ ^65^ ^]^	Mimix
Melt electrowriting	High‐pressure extrusion of fine fibers onto an electrically charged platform.	Ability to print 3D structures using microfilament resolution (>10 µm).^[^ ^66^ ^]^	Novaspider, Regenhu
Projection micro stereolithography	3D printing via layer‐by‐layer projection into a photocurable resin	Mechanical innovations in the machine unlock features as small as 2 *µ*m in photocurable biomaterial resins like GelMA.^[^ ^67^ ^]^	Boston Microfabrication

**Figure 3:**
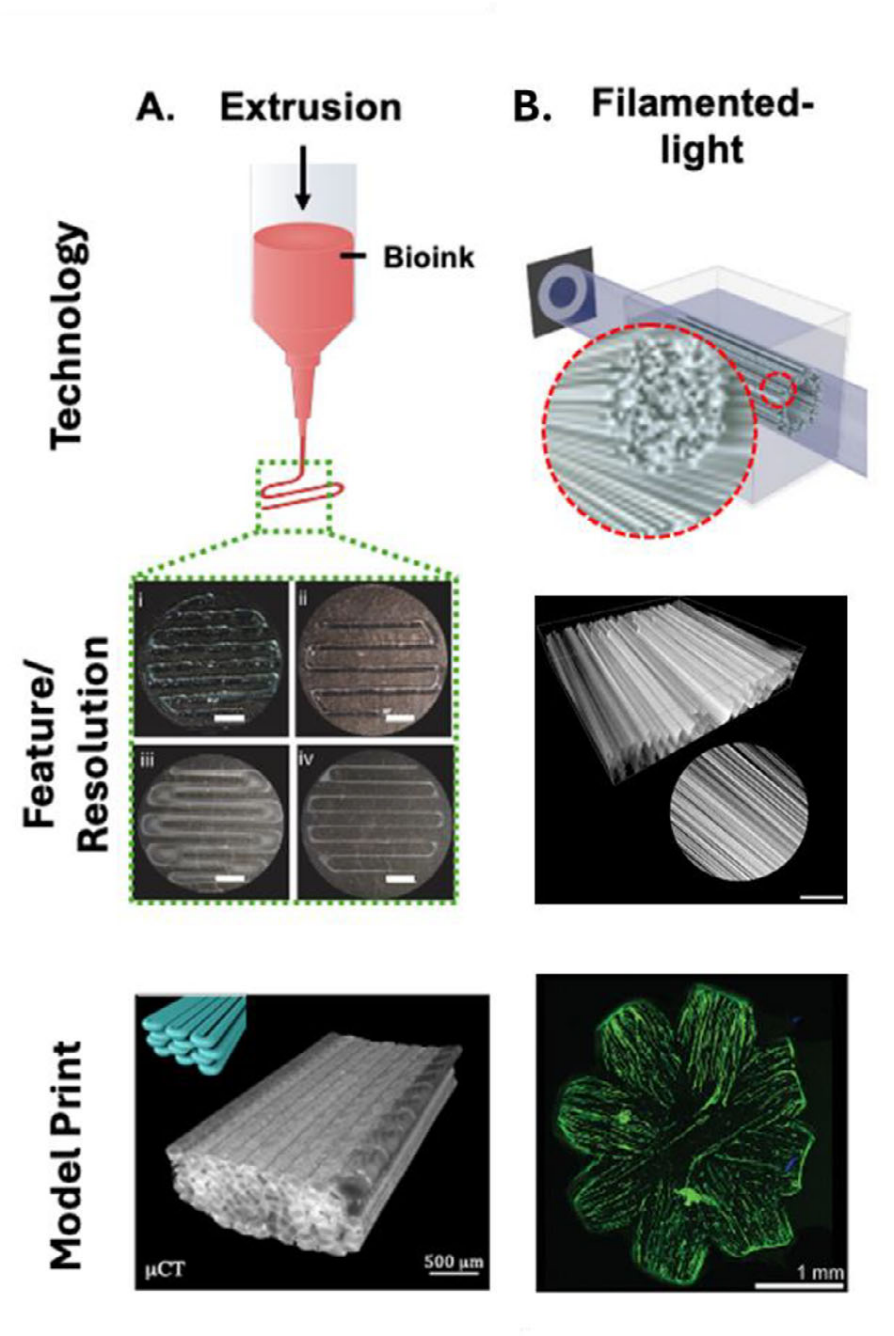
Showcasing the progress in biofabrication resolution. A) A mix of nozzle diameter and bioink fluid dynamics properties limited the resolution of high‐shear extrusion 3D printing systems. These features typically range between 0.1 and 1 mm, depending on the input parameters. B) Flight systems apply an optically modulated light beam onto a light‐curable resin. The instability of the focused beam triggers the extrusion of individual fibres into the bioink. FLight can be used to engineer microscale filaments (diameters as small as 5 µm) and other features that are defined by the resolution of the light. The direction of fibre alignment can be modulated to also guide the direction in which cells grow.Reproduced (Adapted) with permission.^[^ ^69a^ ^]^ Copyright 2021, Springer Nature. Reproduced (Adapted) with permission.^[^ ^69b^ ^]^ Copyright2016, IOP Publishing. Reproduced (Adapted) with permission.^[^ ^69c^ ^]^ Copyright 2017, Elsevier. Reproduced under the terms of the Creative Commons CC‐BY License.^[^ ^70^ ^]^ Copyright 2024, American Chemical Society. Reproduced under the terms of the Creative Commons CC‐BY License.^[^ ^18^ ^]^ Copyright 2022, John Wiley and Sons. Reproduced under the terms of the Creative Commons CC‐BY License.^[^ ^18^ ^]^ Copyright 2024, John Wiley and Sons.

The technologies highlighted in this section have a proven track record in engineering biomimicry, accompanied with an evolution of improved resolution over the years. For example, extrusion‐based bioprinting remains one of the most used techniques in the field and has previously shown to drive cellular alignment in extruded hydrogel fibers via shear force.^[^ ^68^ ^]^ However, the resolution of such fibers is often limited to ≈100 µm (**Figure** **3**A).^[^ ^69^ ^]^ Recent breakthroughs, such as the filamented light (FLight) technique, now allows incorporation of microfilaments within 5–20 µm resolution into hydrogel constructs (Figure 3B), facilitating rapid and uniform cellular alignment within days.^[^ ^18^, ^51^ ^]^ This technique also enables multi‐directional cellular alignment to fabricate larger tissue analogues that are more myocardium‐like.

Many of the ultra‐high resolution biofabrication technologies outlined in Table 1 are light‐activated (e.g., DLP, FLight) fabrication processes. Along with improved hardware, a significant innovation in this space was the deployment of photoinitiators, additives that make the material curable to light, that are activated by light within the visible spectrum and not UV light. The first few versions of these printers were built around using UV light‐based photoinitiators (e.g., Irgacure 2959)^[^ ^71^ ^]^ to cure the bioink used. UV‐light may be cytotoxic and mutagenic to cells, thus making it a poor fit‐for‐purpose within the long‐term product development cycle of biofabrication technologies designed to encapsulate cells into a biomaterial matrix.^[^ ^19^ ^]^ Development of photocurable initiators such as lithium phenyl‐2,4,6‐trimethylbenzoylphosphinate^[^ ^72^ ^]^ (LAP – activated at 405 nm light) and tris(2,2′‐bipyridyl)ruthenium(II) dichloride^[^ ^71^ ^]^ (Ru – activated across the visible light spectrum) allow for rapid photocuring (within seconds of light exposure) and have been successfully used to engineer constructs with high cell densities (within the tens of millions).^[^ ^73^ ^]^ Although these photoinitiated reactions may produce potentially cell‐damaging products such as reactive oxygen species,^[^ ^74^ ^]^ the availability of these visible‐light‐activated photoinitiators and the continuous efforts to bring more cell‐friendly products into the market are a significant step toward more cytocompatible, scalable biofabrication tools.

### 4.2 Pairing Advanced Biomanufacturing with Dynamic Materials to Engineer Advanced Aging Models

**Figure 4:**
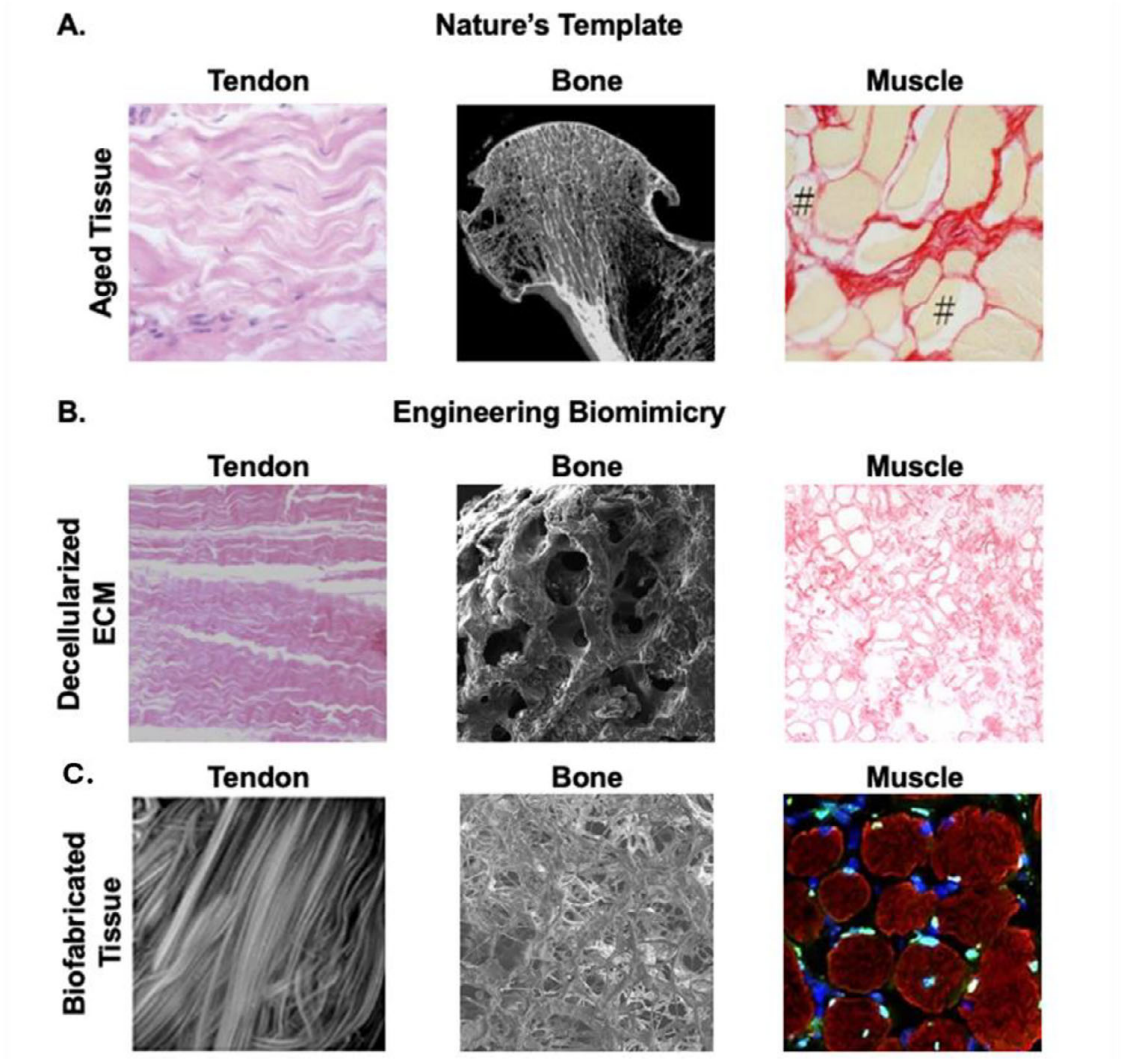
Conceptualising the progression from understanding a natural template to engineering biomimicry. A) Aging is a universal process that impacts each tissue group differently. Tendons lose elasticity and alignment, bone may become more porous, and there is a loss in functional muscle cells (#) and an increase in collagen density with age. B) Decellularized ECM maintains the bulk physical properties of the parent tissue, but there is little to no control over modulating these structures. C) Biofabrication can be used as a tool to engineer physically biomimetic materials that match micro‐topological features of natural tissue. This concept can be extended to engineering the structural features of age using modified bio‐inks such as dECM. Reproduced (Adapted) with permission.^[^ ^32b^ ^]^ Copyright 2019, Elsevier. Reproduced under the terms of the Creative Commons CC‐BY License.^[^ ^79^ ^]^ Copyright 2022, published by MDPI. Reproduced under the terms of the Creative Commons CC‐BY License.^[^ ^80^ ^]^ Copyright 2020, published by Elsevier. Reproduced (Adapted) with permission.^[^ ^81^ ^]^ Copyright 2017, Springer Nature. Reproduced under the terms of the Creative Commons CC‐BY License.^[^ ^44a^ ^]^ Copyright 2018, published in Nature Portfolio. Reproduced under the terms of the Creative Commons 4.0 License.^[^ ^51b^ ^]^ Copyright 2025, IOP Publishing. Reproduced under the terms of the Creative Commons 4.0 License.^[^ ^82^ ^]^ Copyright 2024, The Authors, American Association for the Advancement of Science. Reproduced under the terms of the Creative Commons 4.0 License.^[^ ^83^ ^]^ Copyright 2018, published by Nature Portfolio.

The ultimate criterion for the successful development of new aging models from these technologies will be if workflows can be engineered to fabricate biomimetic healthy young tissue that progressively transforms (independently of the original input technology) into aged tissue, thereby emulating the dynamic microenvironment (architecture and composition) of the aging ECM. The changes in physical features (**Figure** **4**A) include loss of alignment paired with stiffening (e.g., tendon), or increased porosity and brittleness (e.g., bone), or structural disorientation coupled with stiffening (e.g., muscle). Biofabrication technologies enable precise control over such physical features (Figure 4C); however, the utilization of biofabrication for dynamic aging models will depend on the material of choice.

For example, photocurable biomaterials are the only material class compatible with light‐activated bioprinting modalities, such as FLight. Although light‐based fabrication methods are only compatible with light‐curable materials, there is an extensive list of synthetic (e.g., modified polyacrylamide),^[^ ^19^ ^]^ unmodified natural polymers (e.g., silk‐fibroin),^[^ ^14a^ ^]^ and modified natural polymers (e.g., methacrylated hyaluronic acid)^[^ ^75^ ^]^ to choose from. Some of these photocurable materials (e.g., gelatin)^[^ ^76^ ^]^ can be tuned to degrade at different rates. As also highlighted by a recent review from Major et al., light‐activated chemistry has enabled researchers to engineer dynamic systems that physically degrade, stiffen, or soften in response to specific light‐activated stimuli.^[^ ^17^ ^]^ When paired with the right physiologically relevant micro‐morphology, dynamic stiffness and programmed dissolution can act as the physical backbone of new models for aging skeletal muscle (i.e., increasing stiffness), neuronal tissue (i.e., softening and loss of structure), and age‐adjacent diseases such as osteoporosis (i.e., programmed dissolution). Therefore, although the fabrication goals of the studies highlighted in this and other reviews on dynamic biomaterials^[^ ^17^, ^77^ ^]^ mainly focused on disease models and capability building, the same methodologies can conceivably be used to engineer biomimetic aging models that change in response to stimuli or time.

### 4.3 Improving the Biological Relevance of Biomaterial Aging Models With Decellularized ECM

Current in vitro aging models primarily focus on capturing ECM compositional changes, such as using tissue‐specific dECM (Figure 4B), which overlooks the key architectural changes discussed in this paper. This gap in the literature represents an opportunity to engineer hybrid biomaterial models that combine the biological value of dECM with the high‐resolution control of biofabrication. There are multiple ways to integrate dECM into biomaterials or a biofabrication workflow. One recent approach is to encapsulate dECM into a hydrogel, as demonstrated by Sun et. al.^[^ ^78^ ^]^ In their study, Sun et. al. encapsulated decellularized murine cardiac tissue sections from young (1‐2 months) and old (18‐24 months) donors in a polyacrylamide hydrogels tuned to match the stiffness of young (≈10 kPa) and aged (≈40 kPa) tissue. The novel decellularization protocol used to engineer this system (termed DECIPHER), allowed Sun et al. to transfer the core microarchitectural and biological components of cardiac ECM to environments of different bulk stiffnesses. Within an accelerated model time of several days and cardiac fibroblasts as the cell line of choice, researchers demonstrated that although the biological age of dECM was a major driver of cellular senescence, stiffened environments paired with young dECM can trigger cellular senescence. Another potential progression in this space is modifying dECM into aging bioinks^[^ ^44c^ ^]^ for input into 3D bioprinters or other biofabrication technologies. Several studies have utilized dECM bioinks to bioprint scaffolds for tissue engineering and regenerative medicine purposes, but have yet to be adapted for aging studies.^[^ ^44^, ^62^ ^]^ Perhaps the recent progress made with dECM bioinks presents an opportunity to establish novel workflows to engineer aging models that combine the structural tunability of biofabrication with the biomimetic functional representation of the ECM. Furthermore, the dECM can be modified using macromolecular chemistry to impart additional dynamicity over the physical properties of bioprinted aging models.

## 5 Characterizing in Vitro Cellular Senescence as a Hallmark of Aging

The success of 3D in vitro aging models hinges not only on the ability to modulate cellular aging within a practical timeframe but also on robust validation pipelines that can resolve the spatiotemporal effects of the engineered physical and biochemical environment on cellular aging. Aging triggers cellular senescence, a response induced at least in part by telomere shortening over time.^[^ ^84^ ^]^ As such, cellular senescence is one of the recognized hallmarks of aging,^[^ ^85^ ^]^ with well‐established methods for detection and characterization. However, these methods may have to be modified for 3D aging models to account for the influence of material composition and properties on such assays. These validation steps are crucial prerequisites for the implementation of aging models in disease modelling and drug discovery.

### 5.1 Identification of Cellular Senescence

At the heart of organismal aging is a functional decline at the cellular level, termed cellular senescence. Aging cells underpin tissue/organ dysfunction, and the associated impairment of their reparative and/or regenerative activities. Cellular senescence, although defined by permanent cell cycle arrest, is increasingly recognized in both mitotic and postmitotic cells.^[^ ^86^ ^]^ Senescent cells universally lose their ability to respond appropriately to signals, which affects the regulation of a broad range of intercellular and intracellular processes, including cell‐cell communication, proliferation, metabolism, autophagy, and apoptosis.^[^ ^87^ ^]^ The identification of senescence, marked by the progressive loss of these common cell functions, typically involves standardized assays (**Table** **2**). Where relevant, cell‐type‐specific functional tests should also be employed; for example, changes in multi‐lineage differentiation potential can be assayed in senescent progenitor cells such as hematopoietic stem cells via differentiation assays.^[^ ^88^ ^]^ A common functional change that manifests in most, if not all, senescent cell types are changes in the cellular secretome.^[^ ^89^ ^]^). Where relevant, cell‐type‐specific functional tests should also be employed; for example, changes in multi‐lineage differentiation potential can be assayed in senescent progenitor cells such as hematopoietic stem cells via differentiation assays.^[^ ^88^ ^]^ A common functional change that manifests in most, if not all, senescent cell types are changes in the cellular secretome.^[^ ^89^ ^]^ Co‐cultures or cultures with conditioned media are common methods to measure the effects of senescence‐associated signaling that has been linked to accelerated tissue degeneration in aging organisms.^[^ ^89^ ^]^

**Table 2:** Standard assays to detect and quantify functional alterations in senescent cells.

Cellular process	Changes During Senescence	Common Quantification Technique
Apoptosis	Increased resistance^[^ ^90^ ^]^	Viability assays with apoptosis inducers^[^ ^91^ ^]^
Autophagy	Upregulated or downregulated, depending on cell type and senescence trigger^[^ ^92^ ^]^	Autophagic flux assays^[^ ^93^ ^]^
Communication	Pro‐inflammation and invasion^[^ ^94^ ^]^	Assays using co‐cultured cells or conditioned media^[^ ^95^ ^]^
Metabolism	Pro‐glycolytic; decreased OXPHOS efficiency^[^ ^96^ ^]^	Mass spectrometry;^[^ ^97^ ^]^ Seahorse real‐time cell metabolic assays^[^ ^98^ ^]^
Proliferation	Downregulated^[^ ^7^ ^]^	Colony‐forming assay;^[^ ^99^ ^]^ BrdU incorporation^[^ ^100^ ^]^

Quantifying changes to cellular processes and function provides a direct characterization of senescence state, but can be relatively time‐ or resource‐intensive and low‐throughput. As such, extensive efforts have been made to identify molecular phenotypes correlated with cellular senescence, which can be detected with reduced time, labor, cell input, and cost, and detection can be scaled via large‐throughput screenings.^[^ ^101^ ^]^ For example, a standard colony‐forming assay, which measures the senescence‐associated decrease in proliferative potential, requires 14 days of culture, discounting staining time.^[^ ^102^ ^]^ A parallel test in the form of an MTT assay requires three to five days to discern senescence‐induced changes in proliferative function.^[^ ^102^ ^]^ In contrast, Ki67, a nuclear protein shown to be a robust marker of cycling cells,^[^ ^103^ ^]^ was downregulated two days after senescence induction, while senescence‐associated beta‐galactosidase levels increased in 6 h. In addition to time savings, the assays measuring these molecular markers use half the amount of cells required for functional assays.^[^ ^102^ ^]^

Senescence is a sustained stress response that profoundly alters the molecular landscape of cells.^[^ ^5^, ^104^ ^]^ As such, molecular markers used to infer senescence states are primarily related to stress signaling (**Table** **3**). Of note, senescence‐associated beta‐galactosidase assay is one of the most common assays for measuring senescence,^[^ ^105^ ^]^ as the marker robustly responds to a wide range of senescence stressors such as DNA damage and oxidative stress.^[^ ^105b^ ^]^

**Table 3:** Markers of cellular senescence and typical detection methods.

Feature	Sub‐feature	Change during senescence	Quantification techniques
Nuclear changes	Telomere length	Shortened^[^ ^108^ ^]^	Fluorescence in situ hybridization; qPCR; telomere restriction fragment analysis; single telomere length analysis^[^ ^109^ ^]^
	Senescence‐associated heterochromatic foci (SAHF)	Increased^[^ ^110^ ^]^	Fluorescence staining^[^ ^111^ ^]^
	Gamma H2AX	Increased^[^ ^112^ ^]^	Immunostaining; Western blot^[^ ^113^ ^]^
	Ki67	Decreased^[^ ^104^ ^]^	Flow cytometry;^[^ ^114^ ^]^ immunostaining^[^ ^115^ ^]^
	DNA damage response proteins (p16, p21, p53, RB)	Downregulated: RB Upregulated: p16, p21, p53^[^ ^116^ ^]^	qPCR; Western blot^[^ ^45^ ^]^
	LaminB1	Downregulated^[^ ^117^ ^]^	Western blot;^[^ ^117^ ^]^ immunostaining^[^ ^118^ ^]^
	Morphology	Polypoidal, enlarged, decreased convexity, increased aspect ratio^[^ ^119^ ^]^	Microscopy^[^ ^119b^ ^]^
Cytoplasmic changes	Lipofuscin	Upregulated^[^ ^120^ ^]^	Flow cytometry^[^ ^121^ ^]^
	Senescence‐associated beta‐galactosidase	Increased^[^ ^122^ ^]^	Cytochemical or fluorescence staining^[^ ^105b^ ^]^
	Morphology	Increased cytoplasmic content and cell size^[^ ^123^ ^]^	Microscopy;^[^ ^124^ ^]^ flow cytometry^[^ ^125^ ^]^
Extracellular changes	Senescence‐associated secretory proteins	Increased^[^ ^126^ ^]^	Proteomics;^[^ ^127^ ^]^ ELISA;^[^ ^128^ ^]^ cytokine array^[^ ^129^ ^]^

Techniques to measure senescence features in cells are typically chosen with considerations of cell requirement, reagent and instrument availability, multiplexing options, and the end state of assayed cells. For example, fluorescence in situ hybridization to probe telomere length can be performed with fewer cells, compared to molecular techniques such terminal restriction fragment analysis, which require thousands to millions of cells.^[^ ^106^ ^]^ Molecular techniques typically need a certain threshold of yield and purity of subcellular components. In some cases, specialized instruments and skills may be required for such assays, such as mass spectrometers for proteomics studies and flow cytometers for surface marker detection, which may not be readily accessible. Nevertheless, there are often alternative methods that target the same senescence features, albeit at a lower resolution and/or with a more focused scope, such as Western blotting for protein quantification and immunostaining for surface marker detection. Furthermore, correlating cellular senescence states to molecular pathways activated by material properties may be of interest when evaluating in vitro aging models. Mechanosensing pathways relevant to cell‐material interactions, including YAP/TAZ and PIEZO1 proteins, have been linked to senescence.^[^ ^107^ ^]^ Methods that allow concurrent measurement of multiple features in a single cell or within a cell population, such as multiplexed imaging and qPCR, may provide opportunities to map senescence pathways regulated by in vitro 3D aging models. Lastly, depending on the downstream application of the cells, non‐destructive assays that preserve cell viability, such as flow cytometry and live‐cell imaging, may be preferable over methods where cells are either lysed or fixed.

Nevertheless, these senescence markers can manifest variably, depending on cell type,^[^ ^130^ ^]^ inductive agent (e.g., oxidative stress, chemical treatment, irradiation, replicative exhaustion), or the tissue origin of the senescent cells.^[^ ^131^ ^]^ Furthermore, the appearance of these individual markers may not be unique to senescence.^[^ ^132^ ^]^ There is an ongoing need to define a more comprehensive temporal blueprint of senescence markers.^[^ ^131^ ^]^ Furthermore, the appearance of these individual markers may not be unique to senescence.^[^ ^132^ ^]^ There is an ongoing need to define a more comprehensive temporal blueprint of senescence markers. Recently, cellular processes such as the stalling of RNA elongation,^[^ ^133^ ^]^ or novel biomarkers such as MIF,^[^ ^9^ ^]^ have also been reported in association with cellular ageing.

The lack of a single definitive hallmark for senescence makes it imperative to measure multiple molecular markers and cellular functions to define the senescence state of cells.^[^ ^132^ ^]^ Databases such as the Human Ageing Genomic Resources compile genes, drugs, and mutations associated with longevity and aging, based on integrated functional genomics, network analyses, systems biology, and evolutionary analyses. These tools provide a useful resource to mine senescence marker panels specific to the intended cell type or model application.^[^ ^134^ ^]^

## 6 Considerations in Assaying Senescence Markers in 3D Models

Methods for assessing cellular senescence often require direct access to cells or cellular contents. Consequently, senescence assays are frequently conducted on cells cultured as 2D monolayers on TCP. Performing these assays becomes increasingly complex as we transition into 3D biomaterial‐based in vitro models. Interference from the material and the 3D physical environment can make cell‐based experiments challenging to execute effectively. Depending on the material and fabrication method used, potential challenges associated with characterizing cellular senescence include material opacity, nonspecific reactivity with assay reagents, and cellular accessibility. Although characterizing senescence in 3D systems will not be as straightforward as TCP models, there are actionable steps that can be taken to overcome these challenges.

### 6.1 Improving Spatial Imaging and 3D Analysis

Microscopy‐based assays, such as immunostaining for senescence markers or dye‐based differentiation assays, can be hindered by the opacity or optical clarity of the material. Even if the base material is fully transparent, engineering microfeatures or topology into these systems can alter the material's optical clarity. An example is the introduction of aligned fibrous hydrogels engineered using FLight, whereby the newly‐formed fibers may change the final opacity or transparency of the material.^[^ ^18^ ^]^ If optical clarity or opacity impedes microscopy analysis, tissue clearing protocols that shift the refractive index of a material to become more transparent can be applied.^[^ ^135^ ^]^ These protocols can range from intensive incubation and washing steps using harsh detergents^[^ ^136^ ^]^ to simple, minimally cytotoxic reagents that will clear a material with a single incubation step.^[^ ^137^ ^]^ How well these protocols work will depend on the base material and will therefore depend on the system under study. Biomaterial clearing will also aid with other popular microscopy‐based tools, such as multi‐photon microscopy and optical coherence tomography, which may be applied to cell‐laden 3D models.^[^ ^138^ ^]^

### 6.2 Cell Accessibility

Similar to gradients in nutrient and gas delivery,^[^ ^139^ ^]^ cells within 3D materials may display region‐specific, differential accessibility to assay reagents. Therefore, the uniformity of reagent delivery must be carefully tested and optimized in new 3D models to ensure that outcomes are not due to differences in stain or reagent diffusion rates. This consideration is particularly important in models that recapitulate heterogenous environmental signals to give rise to spatially distinct responses. For example, stiffness gradients induce variable differentiation responses in stem cells,^[^ ^140^ ^]^ while cell proliferation and migration can be influenced by spatially determined chemotactic gradients.^[^ ^141^ ^]^ The permeability of gels and scaffolds is increasingly recognized as important for cell viability and function, and has been improved by engineering microchannels and pores.^[^ ^142^ ^]^ These designs may also aid the distribution of assay reagents to preserve senescence features that are spatially defined, although appropriate controls are needed to validate results. Alternatively, cells may be liberated from the 3D models via enzymatic or chemical means, then assayed in bulk separately from the material.^[^ ^143^ ^]^ This approach guarantees cell accessibility to reagents and may be particularly useful when harvesting sub‐cellular components such as RNA, to increase both purity and yield of target molecules for downstream molecular assays.

### 6.3 Material Reactivity

Regardless of material origin, possible interactions between the material and assay components must be accounted for. For instance, natural polymers such as collagen or elastin are often derived from xenogeneic sources.^[^ ^144^ ^]^ When investigating senescence features related to protein production,^[^ ^40b^ ^]^ the selected method, such as mass spectrometry, should be able to distinguish between peptides/proteins from the material and from the encapsulated cells.^[^ ^145^ ^]^ Similarly, synthetic polymers can give rise to degradation products, which may have a biological impact on cells during culture.^[^ ^146^ ^]^ For example, the polymer polylactic acid (PLA) is degraded to lactic acid;^[^ ^146^ ^]^ this resulting decrease in pH may not only affect cell viability during culture, but also confound findings from reactions that are pH‐sensitive, such as the senescence‐associated beta‐galactosidase assay.^[^ ^105b^ ^]^ Furthermore, lactic acid is recognized as a senescence‐associated metabolite,^[^ ^147^ ^]^ suggesting caution with assays that measure metabolic alterations in cells contained on or within PLA materials. Alternatively, recent advancements in radioisotope labelling of small molecules may be used to distinguish the origin of detected lactic acid.^[^ ^148^ ^]^ In general, to understand the contributions of physical features to cellular senescence in 3D in vitro models, assay methods must be optimized based on an understanding of material properties to delineate background material‐derived signals from genuine cellular outputs.

Ultimately, these challenges are not unique to 3D cell‐laden materials and may be addressed with solutions adapted from tissue‐based assays. Although requiring additional optimization and controls, senescence assessment methods already developed for monolayer cultures can be adapted to 3D models for a more holistic understanding of the interplay between material properties and cellular aging.

## 7 Future Applications and Implications of More Biomimetic in Vitro Aging Models

### 7.1 In vitro Aging Models for Senotherapeutics

#### 7.1.1 State of the Art in Senotheraputics

Small molecules that target senescent cells to alleviate age‐related conditions are called senotherapeutics.^[^ ^149^ ^]^ There are two classes of senotherapeutics based on their mechanism of action: senolytics and senomorphics.^[^ ^149^ ^]^ Senolytics refer to therapeutics that eliminate senescent cells to reduce the senescence burden on the body by specifically targeting cells with senescent traits and inducing cell death, termed senolysis.^[^ ^150^ ^]^ Senomorphics seek to suppress the secretory phenotype of senescent cells, thereby mitigating their deteriorative effects on tissue function.^[^ ^151^ ^]^Although the first senolytics were only reported ten years ago,^[^ ^152^ ^]^ more than twenty senotherapeutic clinical trials have been completed or are underway,^[^ ^153^ ^]^ primarily dominated by a combination of the first‐generation senolytics, Dasatinib and Quercitin. The first clinical trials targeted diseases associated with a large senescence burden, such as idiopathic pulmonary fibrosis and diabetic kidney disease.^[^ ^154^ ^]^ Initial trial outcomes have been mostly positive, although one trial was terminated after failing to meet the primary end point.^[^ ^87a^ ^]^ Their long‐term safety and general efficacy in delaying aging are still under investigation.^[^ ^155^ ^]^

#### 7.1.2 Applications of in Vitro Models for Senotherapeutic Development

Senotherapeutics in clinical trials typically involve already approved small molecules.^[^ ^155^ ^]^ Drug development usually entails testing in a 2D culture of senescent cells, followed by in vivo experiments in animal models^[^ ^152^, ^156^ ^]^ and human clinical trials. However, following this standard pipeline, as high as 90‐95% of screened drugs fail to successfully translate in human clinical trials.^[^ ^157^ ^]^ A major contributing factor is thought to be the lack of accurate models of human tissue environments,^[^ ^158^ ^]^ which elicit cell responses to drugs that are different to those observed in vivo.^[^ ^157^, ^158^ ^]^

In vitro human models that recapitulate the native cell environment, including features such as physicality and spatially‐organized cell types,^[^ ^159^ ^]^ are proposed to enable more accurate prediction of in vivo drug responses.^[^ ^158^, ^160^ ^]^ For example, substrate stiffness is shown to affect cell response to chemotherapeutics such as cisplatin and paclitaxel,^[^ ^52^, ^161^ ^]^ and this response varies with cell lines.^[^ ^161^ ^]^ Thus, aging models, in which cells are exposed to physical cues of stiffness, alignment, and porosity that emulate their native aging niche, may better simulate the response of senescing cells to senotherapeutics. When combined with patient‐derived cells such as iPSCs, these models can be used to test patient‐specific responses to drugs, such as senolytics for cardiac aging.^[^ ^162^ ^]^ Such personalized platforms are also beneficial for modelling individual geriatric conditions, given the broad range of age‐related etiologies and patient responses to senotherapeutics.^[^ ^163^ ^]^ Ultimately, these models may provide a functionally superior, more ethical, and more sustainable platform for drug screening and disease modelling compared to resource‐intensive animal models, with the potential to revolutionize geriatric care.^[^ ^52^, ^161^ ^]^

### 7.2 Modelling Interdependency of Aging and Extracellular Vesicle Signaling

Extracellular vesicles (EVs) are secreted by cells as a mode of intercellular communication and a disposal pathway for cellular debris. Aging profoundly influences the concentration, size, and cargo content of EVs.^[^ ^164^ ^]^ As an example, EV fractions from the brain of aged mice show increased levels of hyaluronan and chondroitin sulfate proteoglycans, reflecting the compositional changes in the brain ECM during aging.^[^ ^164b^ ^]^ Aging trajectories also alter the RNA content in EVs, with the magnitude of effect dependent on EV tissue origin and RNA class.^[^ ^164a^ ^]^ Non‐coding RNAs, such as tRNA and miRNAs, are particularly susceptible to modulation by age. In these cases, aberrant changes in EV RNA content do not simply herald broader cellular modifications due to age, but also endogenously drive aging features, via alterations to intercellular communication that disrupt homeostatic balance.^[^ ^165^ ^]^ For instance, miR‐29, which is elevated in plasma‐derived EVs with age,^[^ ^164a^ ^]^ is proposed to be a key regulator of aging phenotypes.^[^ ^166^ ^]^ Conversely, EVs isolated from young tissues or organisms have been shown to reverse age‐related dysfunction. Pro‐longevity miRNAs in plasma EVs from young mice have been shown to rejuvenate aged animals by stimulating PGC‐1α expression and improving mitochondrial energy metabolism.^[^ ^167^ ^]^ Similarly, increasing circulating levels of extracellular nicotinamide phosphoribosyltransferase, carried in young EVs, promotes NAD+ biosynthesis in aged mice, and counteracts aging, enhances overall functions, and extends healthspan.^[^ ^168^ ^]^ Senolytic treatment of old mice likewise shifts EV cargo composition and function toward those of a younger phenotype.^[^ ^169^ ^]^ Some miRNAs may also apply reverse‐dysfunction actions in synergistic mechanisms, such as the synergy between miR‐125, miR‐143, miR‐199, and miR‐122, which were shown to drive an antifibrotic response in both mouse and human cardiac fibroblasts in vitro.^[^ ^170^ ^]^

Next‐generation in vitro aging models can both exploit and contribute to our emerging understanding of the interdependent processes of EV signaling and aging. Engineered models that incorporate age‐, sex‐, or tissue‐specific EV cargo (proteins, lipids, RNAs) can be used to study the synergistic or antagonistic interplay between the physical environment and cell‐derived signaling cues on aging. Alternatively, the EV cargo of cells cultured within these models can be evaluated as a complementary biomarker to more fully recapitulate the complexity of ageing.^[^ ^164a^ ^]^ In vitro aging models can also be used to screen the therapeutic efficacy of EVs, such as in the reduction of reactive oxygen species and protection against oxidative stress, which can be harnessed as interventions to combat age‐related disorders or to promote healthy aging.^[^ ^171^ ^]^

### 7.3 Understanding Aging‐Cancer Interactions Through Engineered Systems

While in vitro models cannot fully recapitulate the complex, systemic nature of aging‐related comorbidities, they offer unique opportunities to investigate specific mechanisms by which aged tissue microenvironments influence disease progression. The disproportionate prevalence of cancer in elderly populations reflects, in part, how aging creates increasingly permissive tissue environments for malignant transformation and progression. Senescent fibroblasts within aged stroma actively promote cancer progression through paracrine signaling. Parrinello et al. demonstrated that senescent fibroblasts disrupt normal epithelial differentiation and promote invasiveness in breast epithelial cells, ultimately driving malignant transformation.^[^ ^172^ ^]^ This SASP creates a pro‐inflammatory, matrix‐degrading environment that facilitates cancer cell invasion and metastasis.

The aged ECM itself also becomes increasingly cancer‐permissive through compositional and architectural changes. Yang et al. showed that matrix‐bound vesicles from aged breast tissue significantly increase cancer cell invasiveness compared to young tissue‐derived vesicles, mediated through altered miRNA and cytokine profiles.^[^ ^173^ ^]^ Similarly, aged collagen networks exhibit increased crosslinking and altered fiber organization that can promote cancer cell migration and metastatic seeding. Current cancer research predominantly uses young, healthy matrix environments that fail to recapitulate the aged tissue context where most cancers arise. Engineered aging models that progressively transition from healthy to aged matrix states could provide more physiologically relevant platforms for studying cancer initiation, progression, and therapeutic resistance. Such models could incorporate the temporal evolution of matrix stiffness, senescent cell accumulation, and inflammatory factor gradients that characterize aging tissues.

These aging‐cancer models would be particularly valuable for investigating age‐related therapy resistance, as aged stroma can protect cancer cells from chemotherapy and immunotherapy through both physical barriers and biochemical signaling. By co‐culturing cancer cells with engineered aging matrices, researchers could identify novel therapeutic targets that overcome age‐related treatment resistance and develop more effective strategies for treating cancer in elderly patients.

### 7.4 Understanding Aging‐Disease Interactions Through Engineered Systems

Incorporating features of established age‐related disease models into the more temporally complex aging models proposed in this perspective is a crucial extension to those aging models. For example, a post‐infarct heart consists of three distinct regions: a remote healthy area (soft), a border zone (slightly stiffer), and the scarred, hypoxic region (stiff).^[^ ^174^ ^]^ A recent study by Basara et al. recreated these three regions by extruding different combinations of materials across three separate representative layers.^[^ ^174^ ^]^ Incorporating temporal features (e.g., stiffening from young to aged to infarcted tissue) adds a layer of complexity that can better capture the cellular response to myocardial infarction. Advanced aging‐related disease models may also serve as valuable tools to investigate disease etiology. For example, some molecules or markers, such as trimethylamine N‐oxide (TMAO), a metabolite produced by the liver that is found at higher levels in the midbrain of patients with Parkinson's.^[^ ^175^ ^]^ Along with more complex physical environments, advanced biomanufacturing can also be used to engineer the controlled release of a molecule of interest,^[^ ^17^ ^]^ adding another temporal component to studying the impact specific molecules have on age‐related disease.

The examples listed in this section represent only a small subset of complex aging‐related diseases that can be modeled. Similar to the discussion about different tissue types in section 2, it is important to remember that aging‐related diseases result from a complex mix of biophysiological and biochemical events that are specific to each tissue type or affected area.^[^ ^176^ ^]^ The detailed biochemical and physiological features of these diseases have been thoroughly studied in conditions like osteoarthritis, heart failure, and nonalcoholic fatty liver disease.^[^ ^176a^ ^]^ Therefore, when designing these aging‐related disease models, researchers must be able to prioritize key variables to simplify these systems into attainable models.

## 8 Conclusion and Closing Remarks

The engineering approaches required to create dynamic, biomimetic in vitro aging models have profound implications that extend far beyond studying aging. The advanced biomaterials and biofabrication workflows developed for this purpose will likely become the same foundational technologies that drive new platforms for personalized medicine, accelerate drug discovery, and enable novel regenerative therapies. Furthermore, by elucidating the environmental factors that trigger cellular aging, we can engineer new blueprints for scalable cellular manufacturing, thereby enhancing the longevity and stability of large‐scale cell cultures. As these market‐ready fabrication technologies continue to develop in terms of functionality and resolution, the reproducibility and overall impact of this technology will depend on the quality of the individual components used to build these models. This is doubly true for non‐synthetic components such as naturally derived bioinks (e.g., silk fibroin)^[^ ^177^ ^]^ or animal‐derived bioinks (e.g., gelatin)^[^ ^178^ ^]^ and cell culture products (e.g., Matrigel®,^[^ ^179^ ^]^ fetal bovine serum),^[^ ^180^ ^]^ since the quality and properties of these components are sensitive to variables ranging from environmental factors (e.g., climate and weather), animal feed, or how the component was purified. The successful transition from lab‐bench to translatable, deployable market‐ready models will depend on establishing stable supply chains, scalable good manufacturing processes backed by comprehensive validation frameworks, potentially leveraging AI‐driven tools for high‐throughput analysis, to ensure these complex models are consistent, reliable, and safe.

## Conflict of Interest

The authors declare no conflict of interest.
